# Impacts of systemic inflammation response index on the prognosis of patients with ischemic heart failure after percutaneous coronary intervention

**DOI:** 10.3389/fimmu.2024.1324890

**Published:** 2024-02-19

**Authors:** Meishi Ma, Kang Wu, Tienan Sun, Xin Huang, Biyang Zhang, Zheng Chen, Zehao Zhao, Jiajian Zhao, Yujie Zhou

**Affiliations:** ^1^Department of Cardiology, Capital Medical University Affiliated Anzhen Hospital, Beijing, China; ^2^Capital Medical University, Personnel Department, Beijing, China; ^3^Department of Cardiology, Bengang General Hospital of Liaoning Health Industry Group, Benxi, China

**Keywords:** ischemic heart failure, systemic inflammation response index, percutaneous coronary intervention, prognosis, MACE

## Abstract

**Background:**

Atherosclerosis and cardiovascular diseases are significantly affected by low-grade chronic inflammation. As a new inflammatory marker, the systemic inflammation response index (SIRI) has been demonstrated to be associated with several cardiovascular disease prognoses. This study aimed to investigate the prognostic impact of SIRI in individuals having ischemic heart failure (IHF) following percutaneous coronary intervention (PCI).

**Methods:**

This observational, retrospective cohort study was conducted at a single site. Finally, the research involved 1,963 individuals with IHF who underwent PCI, with a 36-month follow-up duration. Based on the SIRI quartiles, all patients were classified into four groups. Major adverse cardiovascular events (MACEs) were the primary outcomes. Every element of the main endpoint appeared in the secondary endpoints: all-cause mortality, non-fatal myocardial infarction (MI), and any revascularization. Kaplan–Meier survival analysis was conducted to assess the incidence of endpoints across the four groups. Multivariate Cox proportional hazards analysis confirmed the independent impact of SIRI on both the primary and secondary endpoints. The restricted cubic spline (RCS) was used to assess the nonlinear association between the SIRI and endpoints. Subgroup analysis was performed to confirm the implications of SIRI on MACE in the different subgroups.

**Results:**

The main outcome was much more common in patients with a higher SIRI. The Kaplan–Meier curve was another tool that was used to confirm the favorable connection between SIRI and MACE. SIRI was individually connected to a higher chance of the main outcome according to multivariate analyses, whether or not SIRI was a constant [SIRI, per one−unit increase, hazard ratio (HR) 1.04, 95% confidence interval (95% CI) 1.01–1.07, p = 0.003] or categorical variable [quartile of SIRI, the HR (95% CI) values for quartile 4 were 1.88 (1.47–2.42), p <0.001, with quartile 1 as a reference]. RCS demonstrated that the hazard of the primary and secondary endpoints generally increased as SIRI increased. A non-linear association of SIRI with the risk of MACE and any revascularization (Non-linear P <0.001) was observed. Subgroup analysis confirmed the increased risk of MACE with elevated SIRI in New York Heart Association (NYHA) class III–IV (P for interaction = 0.005).

**Conclusion:**

In patients with IHF undergoing PCI, increased SIRI was a risk factor for MACE independent of other factors. SIRI may represent a novel, promising, and low-grade inflammatory marker for the prognosis of patients with IHF undergoing PCI.

## 1 Introduction

Heart failure (HF) is a multifaceted syndromewith significant mortality and hospitalization rates (1). Ischemic heart disease (IHD) is one of the main factors that induce HF. The main reason is that obstructive atherosclerotic plaques of the coronary artery lead to a reduction of coronary artery blood flow, resulting in myocardial ischemia and cell apoptosis (2, 3). Although the prevention and treatment of ischemic heart failure (IHF) has been optimized over the past few years, they still place a huge burden on society and families (4–6). Therefore, it is important to explore relevant prognostic risk factors, especially simple and easily available risk factors, to guide diagnosis and treatment and improve outcomes in patients with IHF.

In recent decades, a large number of studies have confirmed the effect of low-grade inflammation on atherosclerotic formation and thrombosis, as well as the two-way effect of inflammation and various cardiovascular risk factors (7–9). White blood cells are immune cells that play essential roles in inflammatory disorders (10). Previous studies have shown that the components of white blood cells, including neutrophils, monocytes, and lymphocytes, are reliable biomarkers of systemic inflammation related to a higher potential for cardiovascular illness (11–16). Neutrophils have a significant impact on the inflammatory response of atherosclerosis because they can emit large quantities of inflammatory mediators, chemotactic agents, and anaerobic radicals that cause endothelial cell destruction and consequent tissue ischemia (17–22). Simulation of monocytes and their conversion into lipid-filled macrophages is a fundamental process in atherosclerotic lesion formation (23). In contrast, inflammation is regulated by lymphocytes, which may inhibit atherosclerosis (24, 25).

The systemic inflammation response index (SIRI) is calculated by monocyte, neutrophil, and lymphocyte counts and has emerged as a novel and reliable indicator of chronic low-grade inflammation (26, 27). Previous studies have shown that SIRI is closelyassociated with the prognosis of a variety of cardiovascular diseases, such as ischemic stroke and acute coronary syndrome (ACS) (28–31). However, there have been no studies on the correlation between SIRI and patients’ prospects for recovery from IHF after PCI. Therefore, in this study, we investigated the association between SIRI and prognosis in patients with IHF receiving PCI treatment.

## 2 Method

### 2.1 Study population

**Figure 1:**
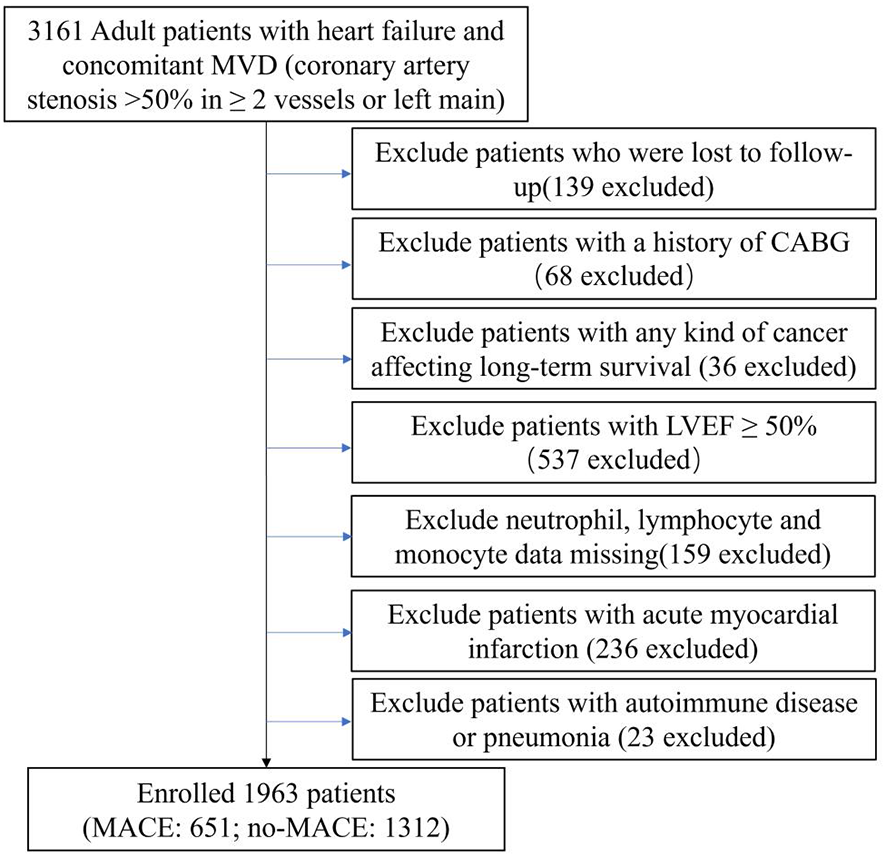
Flow chart of study population. MVD, multivessel disease; CABG, coronary artery bypass grafting; LVEF, left ventricular ejection fraction; MACE, major adverse cardiovascular events.

This research was an exploratory, retrospective cohort investigation conducted at a single site, and we collected data on patients with IHF who underwent selected PCI at Beijing Anzhen Hospital between June 2017 and June 2019. The following criteria were used to determine whether a patient was diagnosed with IHF: (1) HF diagnosis according to ICD (International Classification of Diseases) 10th revision (details can be found in the **Supplementary Material**) and (2) MVD (concomitant multivessel disease: left main artery disease or coronary artery stenosis >50% in >2 vessels) (32). This cohort included 3,161 patients with IHF who underwent elective PCI at our cardiovascular center. The exclusion criteria were as follows: (1) lost to follow-up; (2) history of coronary artery bypass grafting (CABG); (3) any form of tumor compromising long-term survival; (4) left ventricular ejection fraction (LVEF) ≥50%; (5) neutrophils, lymphocytes, and monocytes lacking data; (6) acute myocardial infarction (AMI); and (7) autoimmune disease and pneumonia that may affect white blood cell counts. Finally, 1,963 patients were included in the final study (**Figure 1**).

### 2.2 Data collection and definitions

Demographics, vital signs, New York Heart Association (NYHA) class, diagnosis, comorbidities, history, laboratory parameters, echocardiography, medication use, angiographic data, results of the procedure, and procedural complications were obtained from the electronic medical record system at the Beijing Anzhen Hospital (**Supplementary Material** contains further information). Clinical research coordinators (CRCs) were responsible for accurately inputting these data into an Electronic Data Capture (EDC) system. A minimum of two skilled cardiologists examined the angiographic data. The **Supplementary Material** contains information on the specifics of coronary lesion features. The taxus and cardiac surgery (SYNTAX) score algorithm was used to determine the SYNTAX score (www.syntaxscore.com).

### 2.3 Follow−up

Following baseline PCI, patients underwent regular follow-up evaluations at 3, 6, 9, and 12 months, as well as at intervals of 24 and 36 months by trained healthcare professionals. Major adverse cardiovascular events (MACEs) and medication information were obtained through patient or family visits to the outpatient office or telephone surveys. Under the guidance of professional physicians, CRCs were tasked with overseeing this follow-up process.

MACEs were defined as all-cause mortality, non-fatal myocardial infarction (MI), and any revascularization. The fourth universal definition of MI (2018) was used to define it (33). Coronary revascularization from any cause is the standard definition of revascularization.

### 2.4 Grouping and endpoints

SIRI was determined using the following formula: SIRI was defined as (neutrophils ∗ monocytes)/lymphocytes (34). Patients were divided into four groups according to SIRI quartiles (Quartile 1 [SIRI <0.7944], Quartile 2 [0.7944 ≤SIRI <1.2150], Quartile 3 [1.2150 ≤SIRI <2.0730], and Quartile 4 [SIRI≥2.0730]). MACE served as the primary outcome, and each of its components served as the secondary outcome.

During the follow-up period, if several adverse outcomes occurred, the most serious adverse outcomes (all-cause mortality > non-fatal MI > any revascularization) were included in the analysis. In cases of multiple occurrences, only the initial instance was analyzed. The study continued until June 2022.

### 2.5 Statistical analysis

For continuously distributed data with a normal distribution and reported as mean ± standard deviation (SD), the ANOVA test was employed to compare the performance of the two groups. Median and interquartile range (IQR) were used to portray skewed variables and Kruskal–Wallis tests were used for comparison between groups. Categorical variables were expressed as numbers (percentages) and analyzed using the chi-squared test.

The independent impact of SIRI on outcomes was examined using multivariable Cox proportional hazard models. A hazard ratio (HR) and 95% confidence interval (CI) were shown as the results. Variables in the multivariate regression models were selected using univariate analysis. Multivariate Cox proportional hazard models considered variables with P <0.05 in the univariate analysis. Quartile 1 of SIRI served as the reference group. No variables were included in model I. In model II, age and sex were considered. In model III, age, sex, heart rate, body mass index, NYHA class, prior PCI, lymphocyte count, fasting blood glucose (FBG), albumin, total cholesterol (TC), estimated glomerular filtration rate (eGFR), high-density lipoprotein cholesterol (HDL-C), low-density lipoprotein cholesterol(LDL-C), potassium, uric acid, hs-CRP, left ventricular end-systolic diameter (LVDs), LVEF, angiotensin receptor blocker (ARB), sacubitril valsartan, left main artery (LM) disease, chronic total occlusion, diffuse lesion, in-stent restenosis, SYNTAX score, and the target vessel of LM were incorporated. The incidence of endpoints among the four SIRI quartiles was evaluated using Kaplan–Meier survival analysis and the log-rank test. The restricted cubic spline (RCS) model was used to analyze the nonlinear associations between SIRI and MACE, all-cause mortality, non-fatal myocardial infarction (MI), and any revascularization. The RCS model variables were in line with those of model III. Four knots were selected for analysis based on the Akaike information criterion’s lowest value for the number of knots.

The univariate Cox proportional hazard model was used in the subgroup analysis to investigate the correlation between SIRI and MACE in different subgroups. The P-value for the interaction was computed, and a forest plot was generated.

Stata version 15.0 software (4905 Lakeway Drive, College Station, Texas 77845 USA) and R software (R-project ^®^; R Foundation for Statistical Computing, Vienna, Austria, ver. 4.2.1) were used for statistical analysis. Statistical significance was set at P <0.05.

## 3 Result

### 3.1 Patient characteristics

The median (IQR) SIRI level was 1.215 (range, 0.794–2.073). A total of 1,623 men and 340 women participated in the study. As SIRI quartiles increased, age, white blood cells, neutrophils, mononuclear cells, platelets, FBG, alanine transaminase (ALT), aspartate transaminase (AST), blood nitrogen urea, TC, LDL-C, B-natriuretic peptide (BNP), and hs-CRP values increased significantly; however, systolic blood pressure, lymphocytes, albumin, left atrial diameter, LVDs, left ventricular end-diastolic diameter (LVDd), and LVEF values decreased significantly. Patients in higher quartiles of SIRI presented with more male hypercholesterolemia but less prior MI. Patients in the higher SIRI quartiles received less aspirin and clopidogrel, and more ticagrelor, ezetimibe, beta-blockers, diuretics, loop diuretics, spironolactone, and tovaputan treatment (**Table 1**).

**Table 1:** Characteristics of patients stratified by SIRI quartiles.

Characteristics	Total (n = 1,963)	Quartiles of SIRI				P-value
		Quartile 1 (n = 491)	Quartile 2 (n = 491)	Quartile 3 (n = 491)	Quartile 4 (n = 490)	
Age (years)	60.2 ± 11.0	58.7 ± 11.1	60.2 ± 10.4	60.5 ± 10.5	61.5 ± 11.7	0.001
Sex, n (%)						0.001
Male	1,623 (82.7)	386 (78.6)	397 (80.9)	420 (85.5)	420 (85.7)	
Female	340 (17.3)	105 (21.4)	94 (19.1)	71 (14.5)	70 (14.3)	
Vital signs						
Systolic blood pressure (mmHg)	121.7 ± 18.2	122.8 ± 17.5	123.1 ± 17.2	121.7 ± 19.5	119.3 ± 18.3	0.004
Diastolic blood pressure (mmHg)	73.4 ± 12.1	74.0 ± 11.2	73.7 ± 11.7	73.1 ± 13.4	72.8 ± 12.1	0.432
Heart rate (beats/min)	73.7 ± 11.0	73.3 ± 9.9	72.9 ± 11.9	74.3 ± 10.9	74.3 ± 11.0	0.107
Body mass index (kg/m^2^)	26.4 ± 8.1	26.5 ± 6.9	26.8 ± 8.0	25.6 ± 7.2	26.5 ± 9.8	0.122
NYHA class, n (%)						0.609
I	217 (11.1)	53 (10.8)	62 (12.6)	49 (10.0)	53 (10.8)	
II	1,027 (52.3)	275 (56.0)	244 (49.7)	254 (51.7)	254 (51.8)	
III	652 (33.2)	150 (30.5)	169 (34.4)	171 (34.8)	162 (33.1)	
IV	67 (3.4)	13 (2.6)	16 (3.3)	17 (3.5)	21 (4.3)	
Diagnosis, n (%)						0.913
Stable angina	425 (21.7)	110 (22.4)	102 (20.8)	109 (22.2)	104 (21.2)	
Unstable angina	1,538 (78.4)	381 (77.6)	389 (79.2)	382 (77.8)	386 (78.8)	
Comorbidities, n (%)						
Atrial fibrillation	80 (4.1)	15 (3.1)	22 (4.5)	14 (2.9)	29 (5.9)	0.054
Hypertension	1,124 (57.3)	282 (57.4)	274 (55.8)	291 (59.3)	277 (56.5)	0.718
Diabetes	766 (39.0)	192 (39.1)	210 (42.8)	202 (41.1)	162 (33.1)	0.011
Hypercholesterolemia	1,430 (72.8)	330 (67.2)	352 (71.7)	359 (73.1)	389 (79.4)	<0.001
History, n (%)						
Prior stroke	176 (9.0)	46 (9.4)	37 (7.5)	55 (11.2)	38 (7.8)	0.157
Prior MI	479 (24.4)	167 (34.0)	127 (25.9)	110 (22.4)	75 (15.3)	<0.001
Prior PCI	214 (10.9)	47 (9.6)	49 (10.0)	53 (10.8)	65 (13.3)	0.246
Laboratory parameters						
White blood cell (10^9^/L)	8.0 ± 2.8	6.2 ± 1.5	7.1 ± 1.5	7.9 ± 1.7	10.7 ± 3.7	<0.001
Neutrophil (10^9^/L)	5.5 ± 2.7	3.7 ± 1.1	4.5 ± 1.1	5.5 ± 1.3	8.4 ± 3.4	<0.001
Mononuclear cell (10^9^/L)	0.5 ± 0.2	0.3 ± 0.1	0.4 ± 0.1	0.5 ± 0.1	0.7 ± 0.3	<0.001
Lymphocyte (10^9^/L)	1.8 ± 0.6	2.0 ± 0.6	1.9 ± 0.6	1.8 ± 0.6	1.5 ± 0.6	<0.001
Red blood cell (10^9^/L)	4.5 ± 0.6	4.5 ± 0.5	4.5 ± 0.5	4.5 ± 0.6	4.5 ± 0.7	0.561
Platelet (10^9^/L)	222.4 ± 64.8	210.3 ± 62.5	221.2 ± 60.3	227.2 ± 67.8	230.8 ± 66.5	<0.001
Hemoglobin (g/L)	138.7 ± 18.1	139.2 ± 17.1	139.5 ± 17.3	138.0 ± 18.0	138.0 ± 20.0	0.428
FBG (mmol/L)	7.3 ± 3.0	6.9 ± 2.7	7.2 ± 2.9	7.1 ± 3.0	7.8 ± 3.2	<0.001
Triglyceride (mmol/L)	1.7 ± 1.1	1.8 ± 1.1	1.7 ± 1.1	1.7 ± 1.1	1.68 ± 1.0	0.765
ALT (U/L)	25 (17,40)	22 (15,32)	25 (17,33)	25 (16,38)	33.5 (23,62)	<0.001
AST (U/L)	23 (18,37)	21 (17,26)	21 (17,29)	23 (17,34)	43 (21,174)	<0.001
Albumin (g/L)	41.6 ± 3.9	42.6 ± 3.7	42.1 ± 3.6	41.6 ± 3.7	40.2 ± 4.2	<0.001
Creatinine (μmol/L)	86.6 ± 65.0	80.8 ± 33.5	83.6 ± 52.7	89.5 ± 70.9	92.4 ± 89.1	0.019
Blood nitrogen urea (mmol/L)	6.5 ± 2.9	6.2 ± 2.3	6.4 ± 2.4	6.4 ± 3.1	6.9 ± 3.7	<0.001
eGFR (ml/min/1.73 m^2^)	88.0 ± 20.7	89.9 ± 19.6	88.4 ± 19.4	87.6 ± 21.3	86.0 ± 22.1	0.031
TC (mmol/L)	4.1 ± 1.1	4.0 ± 1.0	3.9 ± 0.9	4.1 ± 1.1	4.4 ± 1.2	<0.001
LDL-C (mmol/L)	2.4 ± 0.9	2.3 ± 0.8	2.3 ± 0.8	2.4 ± 0.9	2.7 ± 1.0	<0.001
HDL-C (mmol/L)	1.0 ± 0.2	1.0 ± 0.2	1.0 ± 0.2	1.0 ± 0.2	1.0 ± 0.3	0.060
Sodium (mmol/L)	139.0 ± 3.0	139.2 ± 3.0	139.1 ± 2.8	139.3 ± 2.8	138.5 ± 3.4	<0.001
Potassium (mmol/L)	4.2 ± 0.5	4.2 ± 0.4	4.2 ± 0.4	4.2 ± 0.5	4.1 ± 0.5	0.108
Uric acid (μmol/L)	369.4 ± 97.8	369.0 ± 95.7	370.3 ± 94.0	366.2 ± 99.5	372.1 ± 102.1	0.819
HbA1c (%)	6.8 ± 1.4	6.7 ± 1.3	7.0 ± 1.5	6.8 ± 1.4	6.7 ± 1.4	0.021
BNP (pg/ml)	336 (149, 485)	271 (108, 450)	313 (140, 452)	346 (153, 507)	379 (211, 544)	<0.001
hs-CRP(mg/L)	2.57 (0.92, 7.86)	1.26 (0.56, 3.03)	2.09 (0.84, 4.19)	2.57 (0.88, 8.62)	7.19 (2.57, 20)	<0.001
Echocardiography						
Left atrial diameter (mm)	39.2 ± 5.2	39.6 ± 5.2	39.6 ± 5.3	39.2 ± 4.8	38.5 ± 5.3	0.004
LVDs (mm)	41.2 ± 8.1	42.8 ± 8.2	42.1 ± 8.3	40.8 ± 7.5	39.2 ± 7.9	<0.001
LVDd (mm)	54.8 ± 7.2	56.4 ± 7.3	55.6 ± 7.3	54.6 ± 6.6	52.8 ± 7.2	<0.001
LVEF (%)	40.4 ± 6.5	40.7 ± 6.2	40.4 ± 6.4	41.0 ± 6.0	39.4 ± 7.4	0.001
Medication use, n (%)						
Aspirin	1,956 (99.6)	491 (100.0)	490 (99.8)	486 (99.0)	489 (99.8)	0.038
Clopidogrel	1,582 (80.6)	430 (87.6)	400 (81.5)	402 (81.9)	350 (71.4)	<0.001
Ticagrelor	380 (19.4)	61 (12.4)	91 (18.5)	89 (18.1)	139 (28.4)	<0.001
Statins	1,949 (99.3)	490 (99.8)	488 (99.4)	487 (99.2)	484 (98.8)	0.289
Ezetimibe	466 (23.7)	95 (19.3)	108 (22.0)	133 (27.1)	130 (26.5)	0.011
Oral anticoagulants	82 (4.2)	17 (3.5)	19 (3.9)	18 (3.7)	28 (5.7)	0.266
Warfarin	37 (1.9)	5 (1.0)	8 (1.6)	8 (1.6)	16 (3.3)	0.060
Factor Xa inhibitors	29 (1.5)	7 (1.4)	9 (1.8)	6 (1.2)	7 (1.4)	0.882
Factor IIa inhibitors	16 (0.8)	5 (1.0)	2 (0.4)	4 (0.8)	5 (1.0)	0.679
CCB	239 (12.2)	62 (12.6)	52 (10.6)	74 (15.1)	51 (10.4)	0.089
Beta-blockers	1,186 (60.4)	296 (60.3)	272 (55.4)	306 (62.3)	312 (63.7)	0.044
ACEI	171 (8.7)	40 (8.1)	32 (6.5)	45 (9.2)	54 (11.0)	0.087
ARB	221 (11.3)	48 (9.8)	65 (13.2)	54 (11.0)	54 (11.0)	0.381
Diuretics	1,314 (66.9)	321 (65.4)	303 (61.7)	326 (66.4)	364 (74.3)	<0.001
Loop diuretics	1,132 (57.7)	262 (53.4)	263 (53.6)	283 (57.6)	324 (66.1)	<0.001
Thiazine diuretics	100 (5.1)	30 (6.1)	25 (5.1)	21 (4.3)	24 (4.9)	0.623
Spironolactone	930 (47.4)	234 (47.7)	202 (41.1)	244 (49.7)	250 (51.0)	0.010
Tovaputan	66 (3.4)	12 (2.4)	9 (1.8)	13 (2.6)	32 (6.5)	<0.001
Sacubitril valsartan	646 (32.9)	160 (32.6)	146 (29.7)	169 (34.4)	171 (34.9)	0.302
Metformin	194 (9.9)	40 (8.1)	52 (10.6)	54 (11.0)	48 (9.8)	0.453
Alpha−glucosidase inhibitor	147 (7.5)	27 (5.5)	39 (7.9)	45 (9.2)	36 (7.3)	0.175
Sulfonylurea	42 (2.1)	13 (2.6)	13 (2.6)	10 (2.0)	6 (1.2)	0.362
Insulin	442 (22.5)	113 (23.0)	110 (22.4)	103 (21.0)	116 (23.7)	0.773
Angiographic data						
LM disease, n (%)	360 (18.3)	86 (17.5)	90 (18.3)	91 (18.5)	93 (19.0)	0.947
Three−vessel disease, n (%)	1,111 (56.6)	270 (55.0)	265 (54.0)	287 (58.5)	289 (59.0)	0.100
Chronic total occlusion, n (%)	541 (27.6)	134 (27.3)	134 (27.3)	135 (27.5)	138 (28.2)	0.989
Diffuse lesion, n (%)	385 (19.6)	104 (21.2)	106 (21.6)	85 (17.3)	90 (18.4)	0.250
In-stent restenosis, n (%)	82 (4.2)	20 (4.1)	15 (3.1)	21 (4.3)	26 (5.3)	0.372
SYNTAX score	21.9 ± 7.8	21.8 ± 7.8	21.7 ± 7.8	21.8 ± 7.9	22.2 ± 7.6	0.741
Procedural results						
Target vessel territory, n (%)						
LM	327 (16.7)	80 (16.3)	78 (15.9)	83 (16.9)	86 (17.6)	0.905
LAD	1,475 (75.1)	376 (76.6)	364 (74.1)	356 (72.5)	379 (77.3)	0.271
LCX	1,258 (64.1)	310 (63.1)	317 (64.6)	311 (63.3)	320 (65.3)	0.879
RCA	1,361 (69.3)	343 (69.9)	329 (67.0)	349 (71.1)	340 (69.4)	0.569
Complete revascularization, n (%)	1,201 (61.2)	308 (62.7)	297 (60.5)	296 (60.3)	300 (61.2)	0.859
Number of stents	3.3 ± 1.5	3.4 ± 1.5	3.3 ± 1.4	3.3 ± 1.5	3.4 ± 1.5	0.291
Procedural complications						
Slow-reflow	15 (0.7)	2 (0.4)	5 (1.0)	4 (0.8)	4 (0.8)	0.735
Dissection	21 (1.1)	4 (0.8)	5 (1.0)	6 (1.2)	6 (1.2)	0.912
Perforation	10 (0.5)	2 (0.4)	4 (0.8)	3 (0.6)	1 (0.2)	0.571

Continuous variables were presented as mean ± SD or median (IQR). Categorical variables were presented as number (percentage). P-values were calculated using analysis of variance, Kruskal–Wallis test or Chi-square test to compare differences in variables between different SIRI quartiles. SIRI, systemic inflammation response index; NYHA, New York Heart Association; MI, myocardial infarction; PCI, percutaneous coronary intervention; FBG, fasting blood glucose; ALT, alanine transaminase; AST, aspartate transaminase; eGFR, estimated glomerular filtration rate; TC, total cholesterol; LDL-C, low-density lipoprotein cholesterol; HDL-C, high-density lipoprotein cholesterol; HbA1c, glycosylated hemoglobin A1c; BNP, B-natriuretic peptide; hs-CRP, high sensitivity C-reactive protein; LVDs, left ventricular end-systolic diameter; LVDd, left ventricular end-diastolic diameter; LVEF, left ventricular injection fraction; CCB, calcium channel blocker; ACEI, angiotensin-converting enzyme inhibitor; ARB, angiotensin receptor blocker; LM, left main artery; LAD, left anterior descending artery; LCX, left circumflex artery; RCA, right coronary artery; SYNTAX, synergy between PCI with taxus and cardiac surgery.

### 3.2 SIRI and endpoints

As shown in **Table 2**, during follow-up, 651 (33.2%) events were documented, including 311 (15.8%) all-cause mortality, 64 (3.3%) non-fatal MI, and 276 (14.1%) any revascularization. As the SIRI quartiles increased, the incidence of MACE (P <0.001), all-cause mortality (P = 0.005), and any revascularization (P <0.001) significantly increased. However, no significant difference in non-fatal MI (P = 0.372) was observed among the four groups.

**Table 2:** Outcomes of patients stratified by SIRI quartiles.

Outcomes	Total (n = 1,963)	Quartiles of SIRI				P-value
		Quartile 1 (n = 491)	Quartile 2 (n = 491)	Quartile 3 (n = 491)	Quartile 4 (n = 490)	
MACE, n (%)	651 (33.2)	115 (23.4)	144 (29.3)	169 (34.4)	223 (45.5)	<0.001
All-cause mortality	311 (15.8)	61 (12.4)	75 (15.3)	74 (15.1)	101 (20.6)	0.005
Non-fatal MI	64 (3.3)	14 (2.9)	14 (2.9)	14 (2.9)	22 (4.5)	0.372
Any revascularization	276 (14.1)	40 (8.2)	55 (11.2)	81 (16.5)	100 (20.4)	<0.001

Categorical variables were presented as number (percentage). P-values were calculated using Chi-square test to compare differences in outcomes between different SIRI quartiles. SIRI, systemic inflammation response index; MACE, major adverse cardiovascular events; MI, myocardial infarction.

**Figure 2:**
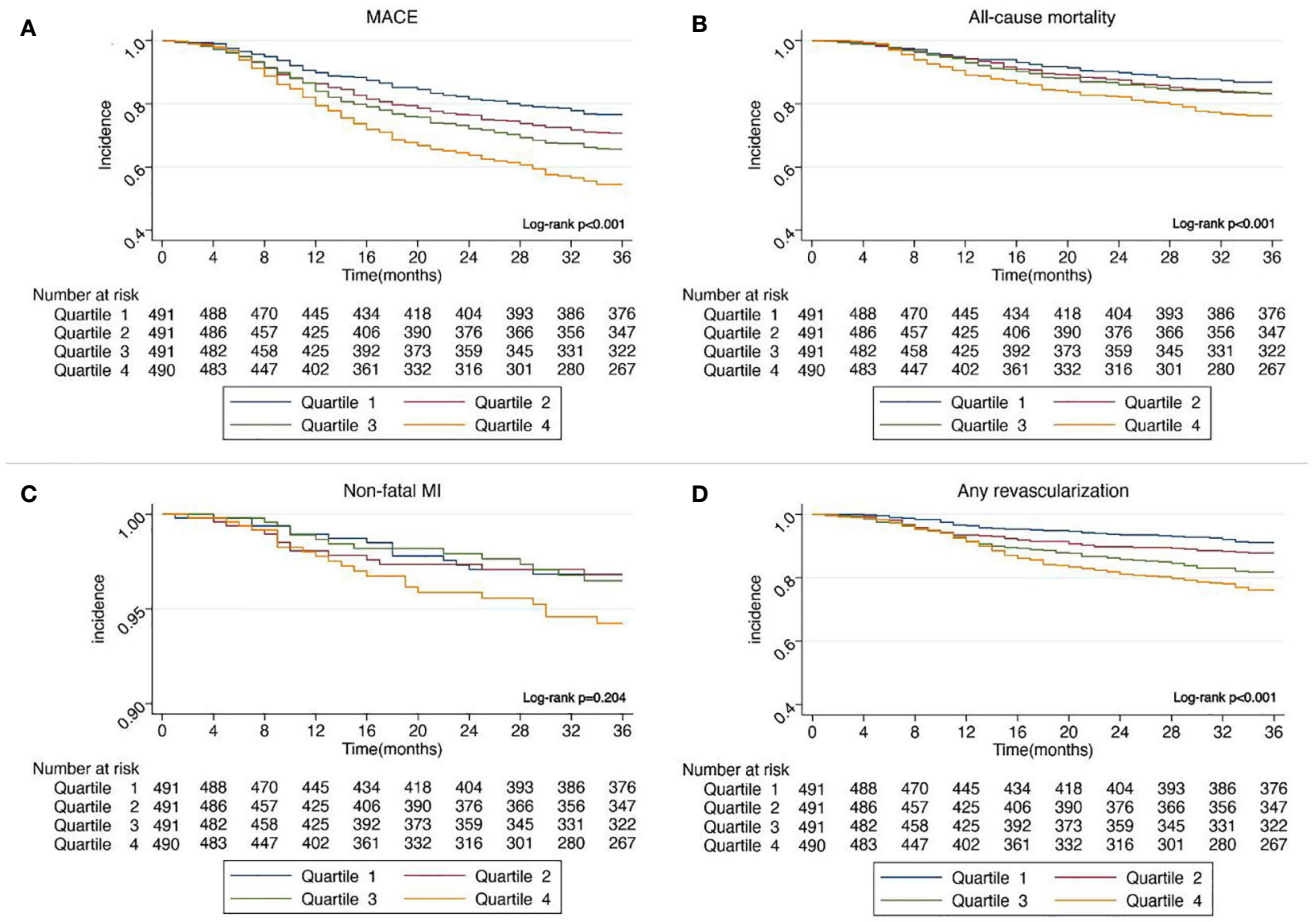
**(A)** Kaplan–Meier curves showing the association between SIRI quartiles and MACE. **(B)** Kaplan–Meier curves showing the association between SIRI quartiles and all-cause mortality. **(C)** Kaplan–Meier curves showing the association between SIRI quartiles and non-fatal MI. **(D)** Kaplan–Meier curves showing the association between SIRI quartiles and any revascularization. SIRI, systemic inflammation response index; MACE, major adverse cardiovascular events; MI, myocardial infarction.

In **Figure 2**, Kaplan–Meier curves for MACE, all-cause mortality, non-fatal MI, and any revascularization stratified by quartiles of SIRI showed that patients with higher SIRI quartiles had a higher incidence of MACE (Log-rank p <0.001), all-cause mortality (Log-rank p <0.001), and any revascularization (log-rank p <0.001). However, no statistically significant difference in non-fatal MI was observed among four SIRI quartiles (log-rank p = 0.204).

The independent impact of SIRI on outcomes was confirmed using Cox proportional hazard models. In Model I, no variable was adjusted; as SIRI quartiles increased, the risk of MACE, all-cause mortality, and any revascularization significantly increased. In Model II, after correcting for age and sex, the outcomes agreed with those of Model I. In Model III, other potential confounding factors were included, and increased SIRI quartiles remained independently linked to a higher risk of developing MACE (Quartile 4 versus Quartile 1: HR, 95% CI: 1.88, 1.47–2.42, P <0.001, P for trend <0.001), all-cause mortality (Quartile 4 versus Quartile 1: HR, 95% CI: 1.61, 1.13–2.28, P = 0.008, P for trend = 0.010), and any revascularization (Quartile 4 versus Quartile 1: HR, 95% CI: 2.48, 1.66–3.70, P <0.001, P for trend <0.001) in patients with IHF. When SIRI was introduced into the multivariate analysis as a continuous variable in Model III, a one-point increase in SIRI was also significantly related to a higher risk of MACE (HR, 95% CI:1.04, 1.01–1.07, P = 0.003) and any revascularization (HR, 95% CI: 1.05, 1.01–1.10, P = 0.008) (**Table 3**).

**Table 3:** The association between SIRI and MACE.

	Model I			Model II			Model III		
	HR (95% CIs)	P	P for trend	HR (95% CIs)	P	P for trend	HR (95% CIs)	P	P for trend
MACE			<0.001			<0.001			<0.001
Quartile 1	1.0 (Ref)			1.0 (Ref)			1.0 (Ref)		
Quartile 2	1.32 (1.03–1.68)	0.028		1.29 (1.01–1.65)	0.041		1.27 (0.99–1.63)	0.0657	
Quartile 3	1.58 (1.25–2.01)	<0.001		1.53 (1.21–1.94)	<0.001		1.52 (1.19–1.94)	0.001	
Quartile 4	2.24 (1.79–2.81)	<0.001		2.15 (1.71–2.70)	<0.001		1.88 (1.47–2.42)	<0.001	
Continuous	1.03 (1.01–1.05)	0.001		1.03 (1.01–1.05)	0.003		1.04 (1.01–1.07)	0.003	
All-cause mortality			<0.001			<0.001			0.010
Quartile 1	1.0 (Ref)			1.0 (Ref)			1.0 (Ref)		
Quartile 2	1.29 (0.92–1.81)	0.136		1.27 (0.90–1.77)	0.173		1.24 (0.88–1.75)	0.219	
Quartile 3	1.31 (0.93–1.84)	0.120		1.25 (0.89–1.76)	0.193		1.27 (0.89–1.82)	0.180	
Quartile 4	1.92 (1.40–2.64)	<0.001		1.82 (1.32–2.51)	<0.001		1.61 (1.13–2.28)	0.008	
Continuous	1.02 (0.99–1.06)	0.123		1.02 (0.99–1.06)	0.170		1.03 (0.99–1.08)	0.150	
Non-fatal MI			0.082			0.146			0.342
Quartile 1	1.0 (Ref)			1.0 (Ref)			1.0 (Ref)		
Quartile 2	1.05 (0.50–2.20)	0.896		1.01 (0.48–2.13)	0.971		1.01 (0.47–2.14)	0.987	
Quartile 3	1.08 (0.51–2.26)	0.844		0.99 (0.47–2.10)	0.988		0.92 (0.42–2.00)	0.834	
Quartile 4	1.81 (0.93–3.55)	0.082		1.66 (0.84–3.27)	0.144		1.50 (0.70–3.14)	0.308	
Continuous	1.03 (0.98–1.09)	0.268		1.03 (0.97–1.10)	0.322		1.04 (0.95–1.14)	0.421	
Any revascularization			<0.001			<0.001			<0.001
Quartile 1	1.0 (Ref)			1.0 (Ref)			1.0 (Ref)		
Quartile 2	1.44 (0.96–2.17)	0.077		1.43 (0.95–2.14)	0.088		1.42 (0.94–2.15)	0.096	
Quartile 3	2.18 (1.49–3.19)	<0.001		2.15 (1.47–3.14)	<0.001		2.11 (1.43–3.13)	<0.001	
Quartile 4	2.89 (2.00–4.17)	<0.001		2.82 (1.95–4.08)	<0.001		2.48 (1.66–3.70)	<0.001	
Continuous	1.04 (1.01–1.06)	0.006		1.04 (1.01–1.06)	0.007		1.05 (1.01–1.10)	0.008	

Models were derived from Cox proportional hazards regression analysis. **Model I**: unadjusted. **Model II**: adjusted for age, sex. **Model III**: adjusted for age, sex, heart rate, body mass index, NYHA class, prior PCI, lymphocyte, FBG, albumin, TC, eGFR, HDL-C, LDL-C, potassium, uric acid, hs-CRP, LVDs, LVEF, ARB, sacubitril valsartan, LM disease, chronic total occlusion, diffuse lesion, in-stent restenosis, SYNTAX score, target vessel (LM). SIRI, systemic inflammation response index; HR, hazards ratio; CI, confidence interval; NYHA, New York Heart Association; PCI, percutaneous coronary intervention; FBG, fasting blood glucose; TC, total cholesterol; eGFR, estimated glomerular filtration rate; HDL-C, high-density lipoprotein cholesterol; LDL-C, low-density lipoprotein cholesterol; hs-CRP, high sensitivity C-reactive protein; LVDs, left ventricular end-systolic diameter; LVEF, left ventricular injection fraction; ARB, angiotensin receptor blocker; LM, left main artery; SYNTAX, synergy between PCI with taxus and cardiac surgery; MACE, major adverse cardiovascular events; MI, myocardial infarction.

**Figure 3:**
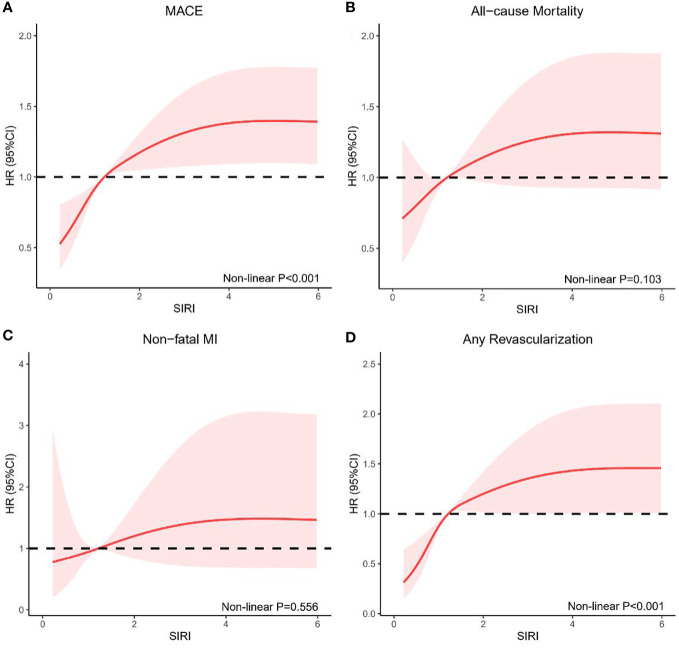
RCS model showing the associations of SIRI with MACE **(A)**, all-cause mortality **(B)**, non-fatal MI **(C)**, and any revascularization **(D)**. RCS, restricted cubic spline; SIRI, systemic inflammation response index; MACE, major adverse cardiovascular events; MI, myocardial infarction; HR, hazards ratio; CI, confidence interval.

The non-linear association of SIRI with the risk of MACE (non-linear P <0.001) and any revascularization (non-linear P <0.001) was confirmed using RCS curves fitted for Cox proportional hazard models. In general, the risk of MACE, all-cause mortality, non-fatal MI, and any revascularization increased as SIRI increased (**Figure 3**).

### 3.3 Subgroup analysis

**Figure 4:**
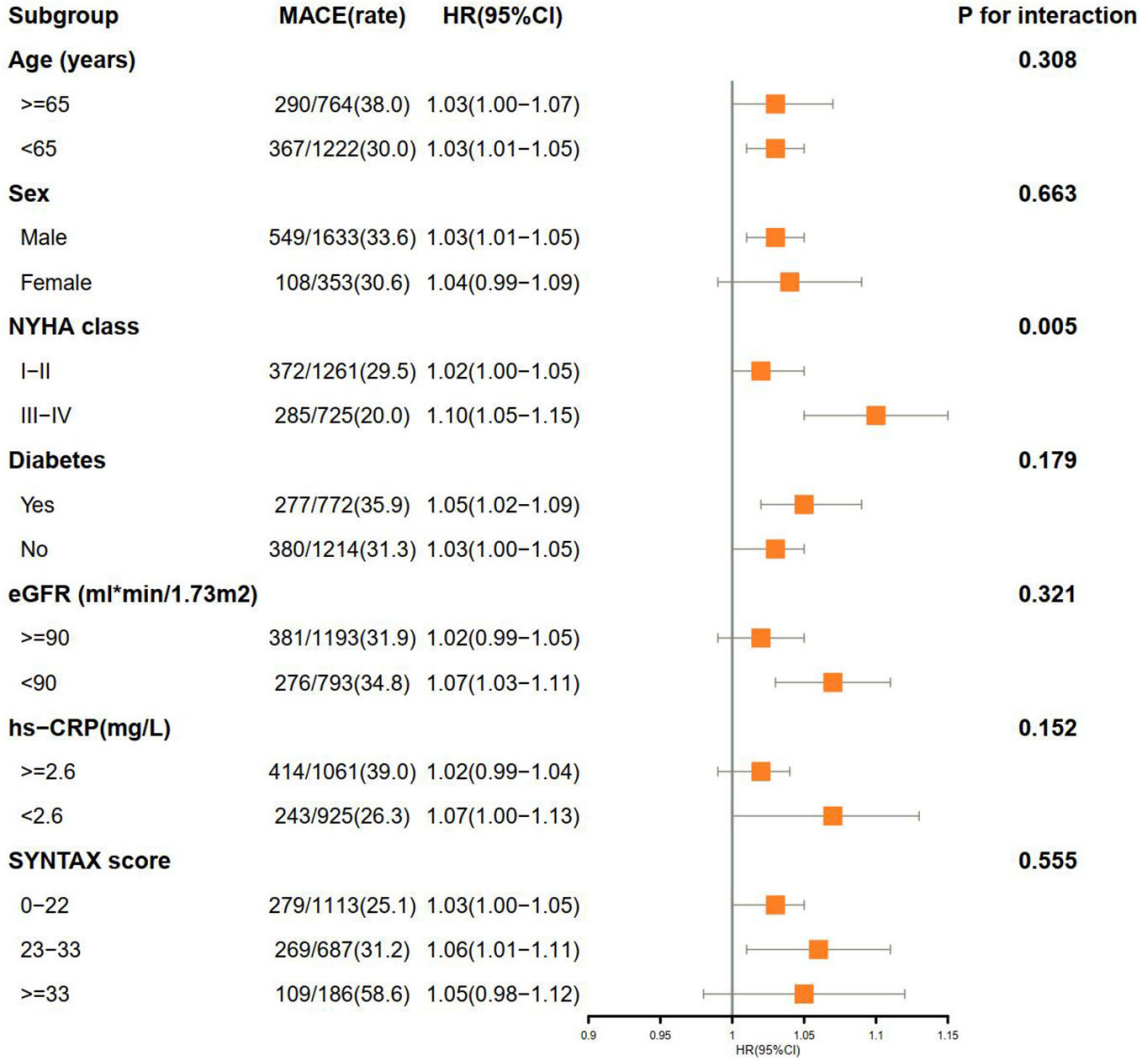
Subgroup analysis of associations between MACE and SIRI. MACE, major adverse cardiovascular events; SIRI, systemic inflammation response index; HR, hazards ratio; CI, confidence interval; NYHA, New York Heart Association; MI, myocardial infarction; eGFR, estimated glomerular filtration rate; hs-CRP, high sensitivity C-reactive protein; SYNTAX, synergy between PCI with taxus and cardiac surgery.

Subgroup analysis confirmed the increased risk of MACE with elevated SIRI in the NYHA class III–IV subgroup (P for interaction = 0.005). No obvious interactions were observed in the other subgroups (**Figure 4**).

## 4 Discussion

The main conclusions of the study are as follows: (1) According to Kaplan–Meier curves, patients with higher SIRI had a higher chance of incidence of MACE, all-cause mortality, and any revascularization. (2) After considering potential confounding variables, SIRI was found to be independently associated with a higher risk of MACE, all-cause mortality, and any revascularization. (3) RCS confirmed a nonlinear relationship between SIRI and MACE and any revascularization. (4) Subgroup analysis confirmed a higher chance of MACE with elevated SIRI in a subgroup of NYHA class III–IV.

The primary contributing factor of IHF is coronary atherosclerosis, which is characterized by narrowing and occlusion of the coronary artery, resulting in insufficient coronary blood flow, myocardial ischemia, and hypoxia (3, 35). The etiology of atherosclerotic disease is heavily influenced by inflammatory processes (36). SIRI has been shown to be an accurate indicator of persistent low-degree inflammation based on monocytes, neutrophils, and lymphocyte counts (27). Previous researches have shown that neutrophils are crucial in the inflammatory response of atherosclerosis by causing apoptosis of small muscle cells to exacerbate vessel wall inflammation, which can secrete significant quantities of inflammatory mediators, chemo chemotactic substances, and anaerobic free radicals to cause endothelial cell damage and subsequent tissue ischemia (17–22). Monocytes are primary cells involved in the development of atherosclerotic plaques. Platelet–monocyte aggregates can form when monocytes stimulate platelets, promoting inflammation, adhesion, and release of vasoactive substances (23). Platelet–monocyte aggregates can also promote thrombosis and blockage of blood vessels, leading to hemodynamic changes (37). Meanwhile, monocytes adhere to the endothelium and differentiate into macrophages (38). Subsequently, by taking up lipids, they become foam cells, activating proinflammatory cytokines and reactive oxygen species release to promote atherosclerosis progression (39). On the other hand, lymphocytes can prevent atherosclerosis and have a regulatory role in inflammation (24, 25). Additionally, prior research has shown a connection between lymphopenia and a poor prognosis in patients with heart failure (HF) and coronary artery disease (CAD) (40–42). Previous studies have shown that higher monocyte, neutrophil, and lymphocyte counts are associated with higher cardiovascular risk (24, 43, 44). Therefore, it is plausible to assume that SIRI, calculated as (neutrophils × monocytes)/lymphocytes, is linked with outcomes in patients with IHF patients following PCI. Furthermore, as a combination of the three, SIRI may amplify the changes in the three.

In recent years, a growing number of studies have found a connection between SIRI and the prognosis of cardiovascular illnesses. A 10-year follow-up period revealed 4,262 stroke occurrences, 1,233 MI events, and 7,225 all-cause deaths in a sizable prospective cohort of 85,154 individuals with cardiovascular disease. The results showed that SIRI was positively associated with stroke (HR, 95% CI:1.194, 1.087–1.313), MI (HR, 95% CI:1.204, 1.013–1.431), and all-cause mortality (HR, 95% CI:1.393, 1.296–1.498) (45). A study conducted by Dziedzic et al. also confirmed a significant association between the SIRI and the CAD severity. ST-segment elevation myocardial infarction (STEMI) individuals had a higher SIRI than stable CAD patients, and a higher SIRI was observed in patients with three-vessel CAD (46). Another study exploring the effect of the inflammation index on the endpoints of ACS patients after PCI also demonstrated that the probability of MACE (HR, 95% CI:3.847, 2.623–5.641) and SIRI were independently associated (31).

This study is the first to explore the role of inflammation assessed using SIRI in the prognosis of patients with IHF after PCI. Consistent with previous findings, similar results from our study showed that SIRI was substantially related to the outcome in patients with IHF receiving PCI. As a traditional inflammatory factor, hs-CRP is widely used in clinical practice (47). In this study, hs-CRP was included in the multivariate Cox regression model, and the results showed that the effect of SIRI on MACE was independent of hs-CRP. Therefore, in clinical practice, we can not only rely on traditional factors to assess the level of inflammation in patients but also pay attention to new inflammatory assessment methods such as SIRI. Subgroup analysis of this study confirmed the increased risk of MACE with elevated SIRI in a subgroup of NYHA class III–IV, which suggested that the role of SIRI should be taken seriously in patients with higher NYHA. Patients with higher NYHA grades often present with more comorbidities (48–50). In patients with heart failure, comorbidities can promote inflammation and lead to myocardial dysfunction through microvascular inflammation, resulting in poor prognosis (51). In this study, patients with a higher NYHA grade had more comorbidities, worse liver and renal functions, and a higher proportion of diabetes, which may be the reason why SIRI had a higher risk of MACE among patients with a higher NYHA grade.

Our study has several limitations. (1) This research was a retrospective cohort investigation conducted at a single facility. The results may be biased due to single-center enrollment, which has to be confirmed in more extensive multicenter randomized controlled studies. (2) Blood cells were tested only once, and their concentrations may have changed during follow-up. A single blood cell count measurement may be affected by additional factors such as particular drugs, which require caution when interpreting the results; (3) All participants in this study were Asians. Whether the results of this study can be generalized to other races requires further study; (4) We have removed patients who may affect SIRI as much as possible, such as malignant tumors (including hematologic malignancies), autoimmune diseases and pneumonia. However, some potential confounders, such as bacterial or viral infections, cannot be removed, which is a research defect.

## Conclusion

As a new inflammatory marker, SIRI is closely related to MACE, all-cause mortality, and revascularization in patients with IHF treated with PCI. The higher the SIRI value, the worse the prognosis. Therefore, SIRI might be a new, potentially low-grade inflammatory measure for predicting outcomes in patients with IHF after PCI.

## Data availability statement

The original contributions presented in the study are included in the article/**Supplementary Material**. Further inquiries can be directed to the corresponding authors.

## Ethics statement

Written or oral informed consent was obtained from each participant, and the study protocol was approved by the Clinical Research Ethics Committee of Beijing Anzhen Hospital, Capital Medical University (2022235X).

## Author contributions

MM: Conceptualization, Data curation, Formal analysis, Investigation, Methodology, Project administration, Writing – original draft, Writing – review & editing. KW: Data curation, Formal Analysis, Investigation, Writing – review & editing. TS: Writing – review & editing. XH: Data curation, Investigation, Writing – review & editing. BZ: Data curation, Investigation, Writing – review & editing. ZC: Data curation, Investigation, Writing – review & editing. ZZ: Investigation, Writing – review & editing. JZ: Conceptualization, Formal analysis, Investigation, Writing – review & editing. YZ: Conceptualization, Formal analysis, Funding acquisition, Resources, Validation, Visualization, Writing – review & editing.
