# Supplementary material for: Impacts of systemic inflammation response index on the prognosis of patients with ischemic heart failure after percutaneous coronary intervention
Source: Front Immunol. 2024 Feb 19;15:1324890. doi: 10.3389/fimmu.2024.1324890 (PMC10910016; doi:10.3389/fimmu.2024.1324890)
Supplement: Supplementary file 1 [file DataSheet_1.pdf]

Supplemental information (1)

1. Ischemic HF patients were diagnosed according to the following criteria:

(1) HF diagnosis according to International Classification of Diseases (ICD) 10th revision I50.106 (left ventricular failure), I50.001 (congestive HF), I50.902 (Cardiac insufficiency), I50.919 (Diastolic heart failure HF), I50.905(Chronic HF) or I50.911 (HF, unspecified)
(2) concomitant multivessel disease (MVD) (coronary artery stenosis >50% in >_2 vessels or left main).

2. The lesion characteristics of the coronary artery were defined as follows:

(1) left main artery (LM) disease: an angiographically estimated stenosis >50% or a fractional flow reserve <0.80 in the left main coronary artery ostium, mid-shaft, or distal bifurcation.
(2) three vessel disease: more than two main coronary branches (vessel diameter ≥ 2 mm) with the extent of stenosis ≥50%.
(3) chronic total occlusion lesion: lesion with complete obstruction [thrombolysis in myocardial infarction (TIMI) flow grade 0] lasting longer than 3 months, which was judged from the previous medical history or coronary angiogram results.
(4) diffuse lesion: a single stenotic lesion with a length of≥ 20 mm. (5) in-stent restenosis: stenosis of ≥50% occurring in the segment inside the stent, 5 mm proximal or distal to the stent.

Supplemental information (2)

Demographics included age and gender. Vital signs included systolic blood pressure, diastolic blood pressure, heart rate and body mass index. Comorbidities and medical history included atrial fibrillation, hypertension, diabetes mellitus, hypercholesterolemia, prior stroke, prior myocardial infarction (MI), and prior PCI. Laboratory parameters included white blood cell, neutrophil, mononuclear cell, lymphocyte, red blood cell, platelet,

hemoglobin, fasting blood glucose (FBG), triglyceride, alanine transaminase (ALT), aspartate transaminase (AST), albumin, creatinine, blood nitrogen urea, estimated glomerular filtration rate (eGFR), total cholesterol (TC), low-density lipoprotein cholesterol (LDL-C), high-density lipoprotein cholesterol (HDL-C), sodium, potassium, uric acid, glycosylated hemoglobin A1c (HbA1c), B-natriuretic peptide (BNP),high sensitivity C-reactive protein (hs-CRP). Concentrations of albumin was quantified by bromocresol green (BCG) method.

Echocardiography data included left atrial diameter, left ventricular end-systolic diameter (LVDs), left ventricular end-diastolic diameter (LVDd), and left ventricular ejection fraction (LVEF). Medication included aspirin, clopidogrel, ticagrelor, statins, ezetimibe, oral anticoagulants, warfarin, factor Xa inhibitors, factor IIa inhibitors, calcium channel blocker (CCB), beta-blockers, angiotensin-converting enzyme inhibitor (ACEI), angiotensin receptor blocker (ARB), diuretics, loop diuretics, thiazide diuretics, spironolactone, tolvaptan, sacubitril/valsartan, metformin, alpha - glucosidase inhibitor, sulfonylurea, insulin. Procedural results included target vessel territory (LM, anterior descending branch (LAD), left circumflex artery (LCX), right coronary artery (RCA)), complete revascularization and number of stents.
